# Colloidal Stability of CA, SDS and PVA Coated Iron Oxide Nanoparticles (IONPs): Effect of Molar Ratio and Salinity

**DOI:** 10.3390/polym14214787

**Published:** 2022-11-07

**Authors:** Siti Nurliyana Che Mohamed Hussein, Zulhelmi Amir, Badrul Mohamed Jan, Munawar Khalil, Azlinda Azizi

**Affiliations:** 1 Department of Chemical Engineering, Faculty of Engineering, University of Malaya, Kuala Lumpur 50603, Malaysia; 2 School of Chemical Engineering, College of Engineering, Universiti Teknologi MARA, Shah Alam 40450, Malaysia; 3 Department of Chemistry, Faculty of Mathematics and Natural Sciences, University of Indonesia, Depok 16424, Indonesia

**Keywords:** colloidal stability, citric acid, iron oxide nanoparticles, PVA, SDS

## Abstract

Iron Oxide Nanoparticles (IONPs) have received unprecedented interest in various applications. The main challenges in IONPs are fluid stability due to agglomeration in a saline condition. This paper aims to investigate the colloidal stability of citric acid (CA), sodium dodecyl sulphate (SDS) and polyvinyl alcohol (PVA) under various molar ratios and levels of salinity. Firstly, the IONPs were synthesized using a facile co-precipitation approach. Secondly, the IONPs were coated using a simple dip-coating method by varying the molar ratio of CA, SDS and PVA. Next, the coated IONPs were characterized by using an X-ray Diffractometer (XRD), Fourier transform infrared spectroscopy (FTIR), and a Field Emission Scanning Electron Microscope (FESEM) for the morphological and crystallographic study of coated IONPs. Finally, the coated IONPs were characterized for their zeta potential value and hydrodynamic size using a Zetasizer and their turbidity was measured using a turbidity meter. It was found that at a low salinity level, 0.07 M of CA-IONPs, a high zeta potential value, a smaller hydrodynamic size, and a high turbidity value of −40.9 mV, 192 nm and 159 NTU were observed, respectively. At a high salinity level, 1.0 M SDS-IONPs recorded a high zeta potential value of 23.63 mV, which corresponds to a smaller hydrodynamic size (3955 nm) and high turbidity result (639 NTU). These findings are beneficial for delivering cutting-edge knowledge, especially in enhanced oil recovery (EOR) applications.

## 1. Introduction

Iron oxide nanoparticles (IONPs) have been extensively used in numerous applications including pharmaceuticals [1], magnetic resonance imaging (MRI) [2], drug delivery [3], biomedical application [4] and in the oil and gas industries [5,6,7,8,9]. This is due to their intrinsic properties such as low cost, low toxicity, small size, high size-to-volume ratio and the fact that they possess superparmagnetism [10]. In the oil and gas sector, the application of IONPs has always been linked to the utilization of magnetic resonance to locate oil wells. However, the application of IONPs in enhanced oil recovery (EOR) has advanced and has been investigated more widely. Shalbafan et al. (2020) and Safaei et al. (2020) demonstrated the ability of IONPs to improve oil recovery via the wettability alteration of sandstone and carbonaceous rock [5,11]. In addition, Khalil and co-workers proved that IONPs can act as a mobility control agent and assist in the formation of structural disjoining pressure [12].

One of the main challenges in utilizing nanoparticles is the stability of IONPs under harsh reservoir conditions. The harsh conditions in the reservoir can hinder the mobility and the ability of the nanoparticles to distribute inside the porous media. The interaction between these magnetic nanoparticles tends to induce aggregation [13]. Typical salinity can range up to >1 M for monovalent and divalent salts and temperatures can reach up to 150 °C [14]. In order to minimize the dispersion stability issues of IONPs in the aqueous medium, a facile approach to surface coat the IONPS with a coating agent was introduced. The surface coating process was introduced to reduce precipitation and agglomeration issues by providing sufficient repulsive interactions that could stabilize the magnetic and the Vvan der Waals attractive forces on the surface of the IONPs [15].

Recently, various types of coating agent have been used to overcome the potential aggregation and instability problem. These coating agents include citric acid (CA) [13,14,15], sodium dodecyl sulphate (SDS) [5,6,16], polyvinyl alcohol (PVA) [7,17], poly(amino acid)s [18] and polyacrylamide (PAM) [12]. According to Cheraghipour, Javadpour and Medizadeh [19], CA was utilized to stabilize the IONPs in a suspension due to its hydrophillicity properties and its ability to provide a site for surface functionalization. SDS, on the other hand, is an anionic surfactant that can form multilayers together with water on the surfaces of the IONPs [20]. In addition, hydrophilic PVA has hydroxyl groups attached to the carbon structure that can further improve the hydrophilic nature of IONPs and their dispersion in the aqueous phase [21]. It has been well established that these coated IONPs have superior characteristics compared with bare IONPs. Nevetheless, an investigation of surface-coated IONPs and the ability of the coating agent to improve colloidal stability in a high saline environment is still lacking in the literature. Previous literature studies mostly focused on the aqueous phase. The dispersion of nanoparticles in a saline condition is difficult because the strong ionic strength of the saline solution offsets the electrostatic interaction between the nanoparticles. A good colloidal stability is necessary to maintain a high surface area of the nanoparticles. When the nanoparticles are well dispersed in the solution, sufficient repulsive interactions between the particles were achieved [19]. Hence, these can prevent further aggregation and obtain a thermodynamically stable colloidal solution.

This paper compares the colloidal stability of different types of coating agents in order to elucidate the effects of the molar ratio of coating agents and salinity on the zeta potential values, hydrodynamic size and turbidity. In addition, a mechanism of the dispersion stability ofeach coating agent, namely CA, SDS and PVA, was subjected to a saline environment and this is also illustrated. The coating agents selected in this study were based on their superior performance in oil recovery via wettability alteration mechanisms and their stability in aqueous suspension. Therefore, this research aims to select the best conditions of surface-coated IONPs which provide the highest colloidal stability in a saline environment. This outcome is very important for the design of optimal coating conditions and for the further development of such nanoparticles in EOR application.

## 2. Materials and Methods

### 2.1. Materials

Iron (III) chloride hexahydrate (FeCl_3_·6H_2_O, R&M Chemicals, Baddi, India), iron (II) chloride tetrahydrate (FeCl_2_·4H_2_O, Sigma Aldrich, St. Louis, MO, USA, purity ≥ 99.0%), ammonium hydroxide (NH_4_OH, R&M Chemicals, 25 wt%), sodium chloride (NaCl, Fisher, purity ≥ 99.91%), polyvinyl alcohol (PVA, Sigma Aldrich) with an average molecular weight (MW) of 89,000 g/mol and a hydrolysis degree of 99%, sodium dodecyl sulphate (SDS, Sigma Aldrich) and citric acid (CA, Sigma Aldrich) were used throughout the experiment. 

### 2.2. Synthesis of Bare Iron Oxide Nanoparticles

**Figure 1:**
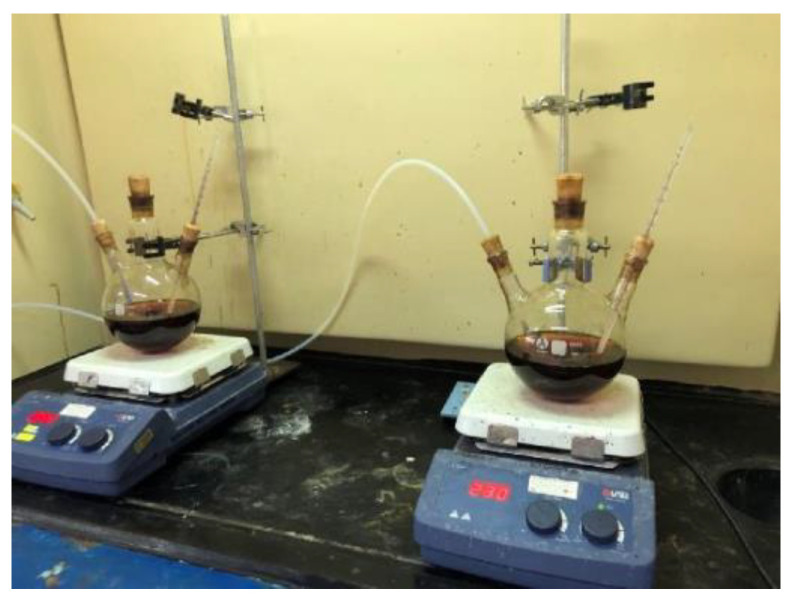
The experimental setup for synthesis of bare iron oxide nanoparticles.

The bare IONPs were synthesized via a facile co-precipitation method [22]. Initially, 12.2 g of FeCl_3_·6H_2_O (0.451 M) and 4.487 g of FeCl_2_·4H_2_O (4.487 M) were added to a three-necked flask filled with 400 mL of distilled water. The mixture was heated to 75 °C and mixed at 500 rpm for 30 min to obtain a homogenous solution. The reaction was carried out under nitrogen gas protection to prevent further oxidation of Fe^2+^ into Fe^3+^. When the mixture turned to a dark-orange colour, ammonium hydroxide was added dropwise to the solution while stirring for 2 h until the pH reached 10. At this stage, the mixture had changed colour to black. The solution was stirred for another 30 min to allow the reaction to be completed and cooled to room temperature. The black precipitate formed was separated by magnetic decantation and washed three times with distilled water to remove any impurities. The precipitate collected was dried in the oven (Universal oven, Memmert) at 60 °C for 12 h. The experimental setup of the synthesis is shown in Figure 1.

### 2.3. Surface Modification of Iron Oxide Nanoparticles with CA, SDS and PVA

CA with 0.1 M was dispersed in distilled water and stirred vigorously at 40 °C for one hour. At the same time, 4.6307 g of bare iron oxide (1 M) nanoparticles were dispersed in 20 mL of distilled water and sonicated using an S80 Elmasonic for 30 min at 600 W. The combination of CA and iron oxide was mixed at a low speed for 20 h at 30 °C to allow the surface modification process to take place. The homogenous solution was then centrifuged using a benchtop centrifuge (3–18 K, Sigma) at 1000 rpm for 15 min to separate the surface-modified iron oxide precipitate from the solution. The process was repeated three times with a series of washes to remove impurities. The precipitate was collected and dried in the oven for 12 h at 60 °C. The surface modification step was repeated for the SDS and PVA in a varying molar ratio (0.07 M, 0.5 M, and 1.0 M). The surface-coated iron oxide nanoparticles for the CA, SDS and PVA will be annotated as CA-IONPs, SDS-IONPs and PVA-IONPs for the following sections of the article for simplicity.

### 2.4. Preparation of CA-IONPs, SDS-IONPs and PVA-IONPs in Saline Solution

Saline solution was prepared by dispersing 177.7 ppm of NaCl in distilled water. 0.1 wt% of CA-IONPs was dispersed in the saline solution and sonicated at 25 °C for 15 min using an ultrasonic bath (Grant XUBA 1, Grant). The same procedure was repeated for SDS-IONPs and PVA-IONPs at 5000 ppm, 17500 ppm, 30000 ppm, 35177 ppm, respectively.

### 2.5. Characterization of CA-IONPs, SDS-IONPs and PVA-IONPs

The crystallite structure of the bare iron oxide nanoparticle, CA-IONPs, SDS-IONPs and PVA-IONPs under various molar ratios was studied using an X-ray diffraction system (ULTIMA IV, Rigaku, Japan) in the range 20° to 80°. The surface morphology of the synthesized samples was examined by field-emission scanning electron microscopy (FE-SEM, JSM-7600 F, JEOL) and the chemical binding of the surface modifiers on iron oxide nanoparticles was analysed using Fourier-transform infrared spectroscopy (FTIR, Spectrum One, Perkin Elmer) at wavelengths between 400 and 4000 cm^−1^. Finally, the zeta potential (Zeta Sizer Nano, Malvern) was used to measure the hydrodynamic size and zeta potential value of CA-IONPs, SDS-IONPs and PVA-IONPs under various molar ratios and salinity ranges. The turbidity of the synthesized samples was also measured using a turbidity meter (2100Q, Hach).

## 3. Results

Different types of chemical characterization techniques were conducted to determine and confirm the properties of the prepared nanoparticles in this work.

### 3.1. Morphological and Crystallographic Analysis

**Figure 2:**
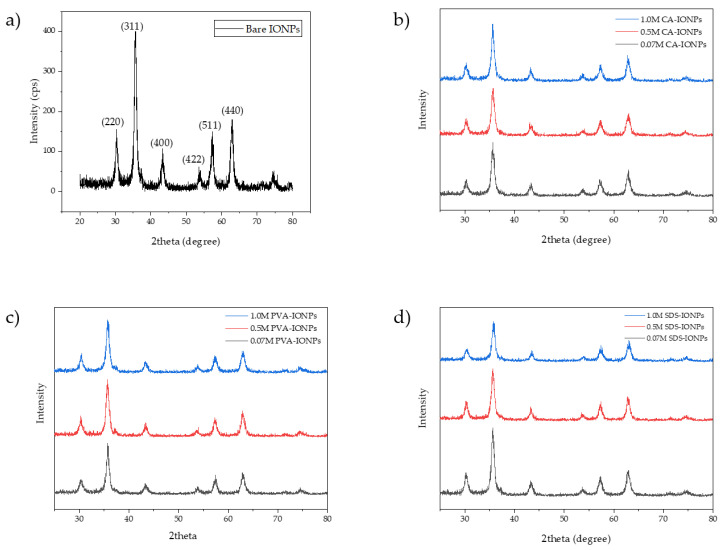
XRD peaks of (**a**) bare IONPs and various molar ratios of (**b**) CA-IONPs, (**c**) PVA-IONPs and (**d**) SDS-IONPs.

The crystallographic structure and morphology of the synthesized nanoparticles were determined by XRD and FE-SEM, respectively. Based on the obtained XRD results in Figure 2a, the crystal structure of bare IONPs is cubic with a space group Fd-3m (227) and lattice parameters of a = b = c = 8.3480 Å. The peaks were in line with the standard XRD patterns of iron oxide (Fe_3_O_4_) (ICDD Card No 01-088-4625) with the angles of 2θ = 30.32°, 35.59°, 43.27°, 53.58°, 57.37° and 62.79° corresponding to (220), (311), (400), (422), (511), and (440) crystalline planes of Fe_3_O_4_ nanoparticles, respectively [5]. Because there no additional peaks were formed, it can be confirmed that the nanoparticles were successfully synthesized [22]. Figure 2b–d showed the peak intensity for CA-IONPs, SDS-IONPs and PVA-IONPs under various molar ratios, respectively. According to a previous study by Nadeem et al. (2016), the broadening of XRD peaks after the IONPs were coated was due to a reduction in the crystallite size and crystallinity of the complexes [23]. However, in this study it is believed that the broadening of peaks could be due to the concealing effect of the coating agents from the IONPs. The bare IONPs and other coated IONPs showed conformable peaks presenting semi-crystalline IONPs in terms of the surface morphology. The semi-crystalline was due to the combination of narrow (crystalline) peaks and broader (amorphous) peaks of the coating agents CA, PVA and SDS on the surface of the IONPs.

**Figure 3:**
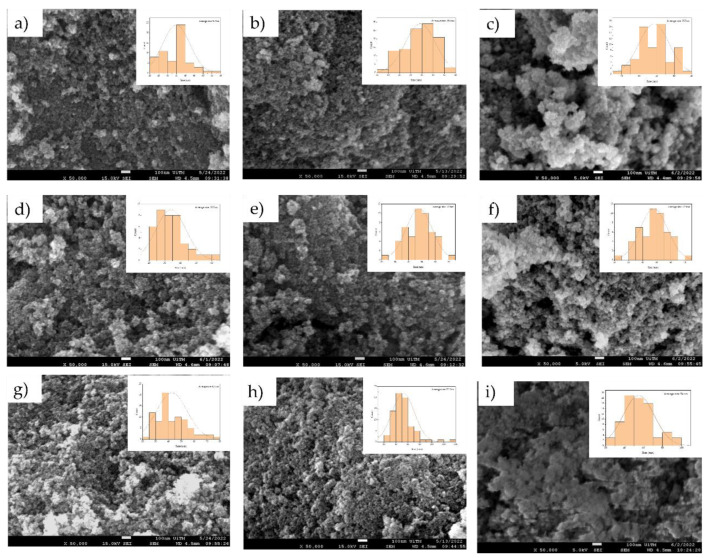
FE-SEM images of (**a**) 0.07 M CA-IONPs, (**b**) 0.07 M PVA-IONPs, (**c**) 0.07 M SDS IONPs, (**d**) 0.5 M CA-IONPs, (**e**) 0.5 M PVA-IONPs, (**f**) 0.5 M SDS-IONPs, (**g**) 1.0 M CA-IONPs, (**h**) 1.0 M PVA-IONPs and (**i**) 1.0 M SDS-IONPs.

The crystallite size of the bare iron oxide nanoparticle, CA-IONPs, SDS-IONPs and PVA-IONPs under various molar ratios were obtained from the XRD results by applying the Scherrer equation:

$$d=\frac{k\lambda }{Bcos\theta }$$

where *d* is the crystallite size in the nanometer, *K* is the Scherrer constant (0.9), *λ* is the wavelength of the X-ray source (0.15406), *β* is the full-width half maximum (FWHM) in radians and *θ* is the peak position in radians. Figure 3 shows FESEM images and the particle size distribution (inset of figure) of synthesized IONPs. Particles with sizes below 100 nm and clusters of variable sizes can be observed for three types of coating agents under different molar ratios. Under increasing molar ratios, the particles seem bigger and exhibit a quasi-spherical smooth surface. Figure 3c,f,i display larger agglomerates; hence, the greatest in particle size at 1.0M of CA-IONPs, SDS-IONPs, and PVA-IONPs. However, under the lower molar ratio of 0.5M CA-IONPs, SDS-IONPs, and PVA-IONPs, the samples showed a better dispersion of the particles on the IONPs surface (Figure 3b,e,h). The irregularities on the surfaces of 1.0 M CA-IONPs, SDS-IONPs, and PVA-IONPs may be attributed to an excess of the coating agent. These results indicate that the presence and amount of coating agents played an important role in the morphology of the synthesized samples. The smallest particle size and least agglomerated can be observed at 0.5 M of SDS-IONPs and 0.5 M of CA-IONPs at 33.9 nm. The particle size distribution is shown in the inset right corner of Figure 3a–i. The average crystallite size of the synthesized nanoparticles and the size measured from FE-SEM images were tabulated in Table 1.

**Figure 4:**
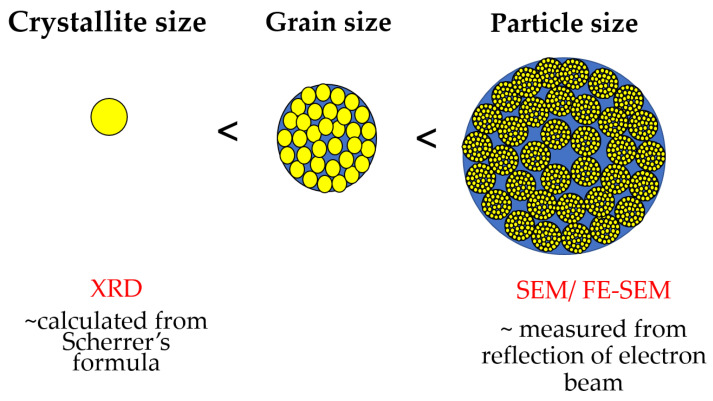
Comparison between crystallite, grain and particle size.

Figure 4 shows the comparison in size between the crystallite size, grain size and particle size. The X-ray peak broadening obtained from XRD was used to evaluate the crystallite size and lattice strain by Williamson-Hall (W-H) analysis. However, the mean particle size of the synthesized nanoparticles was estimated from the FE-SEM results. The crystallite size was generally not the same and usually smaller compared with the particle size due to the presence of polycrystalline aggregates [24].

**Figure 5:**
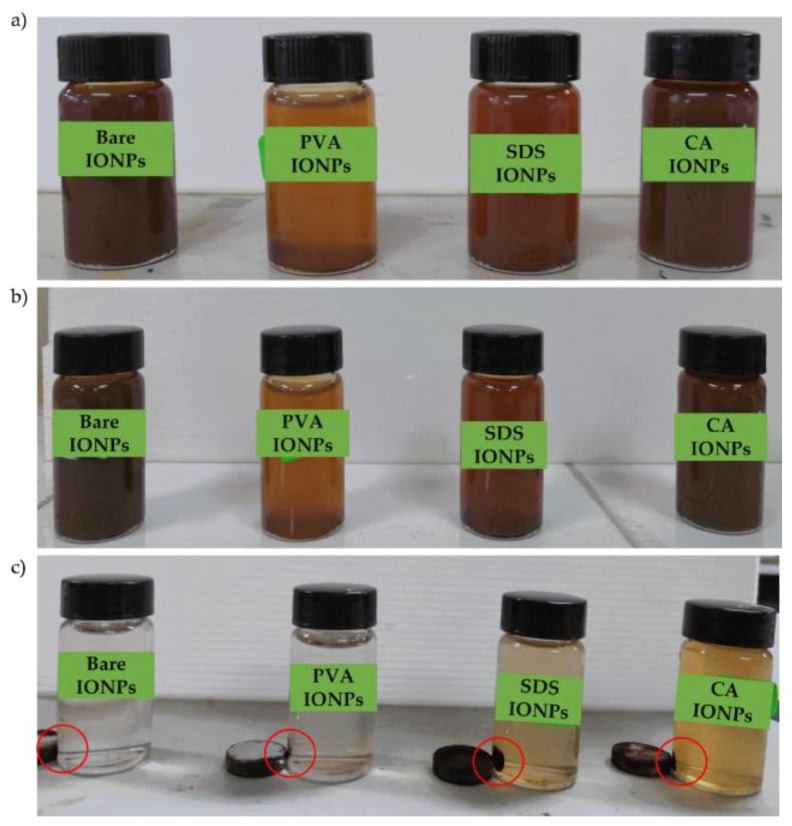
Photograph of aqueous suspensions of bare IONPs, PVA-IONPs, SDS-IONPs, and CA-IONPs after (**a**) 10 min, (**b**) 24 h and (**c**) separation of the nanoparticles using a permanent magnet.

Based on the result in Figure 5a, it is evident that all nanoparticles can be well dispersed in water and exhibited no signs of severe particle agglomeration. It was also observed that after a period of 24 h, no sedimentation occurred (Figure 5b). The observations also agree with the results reported by Khalil and co-workers [12], stating that high colloidal stability is vital in various oil and gas applications as it can maintain the surface area of the nanoparticles. Interestingly, a superparamagnetism state allows the particles to avoid rapid agglomeration in the absence of external magnetic fields due to their zero coercivity (H_c_) [12]. When a single domain magnetism was applied, all of the magnetic spin was in the same direction [25]. This can be seen in Figure 5c, as all the nanoparticles are easily separated from the suspension using a permanent external magnet. 

### 3.2. FTIR Analysis

**Figure 6:**
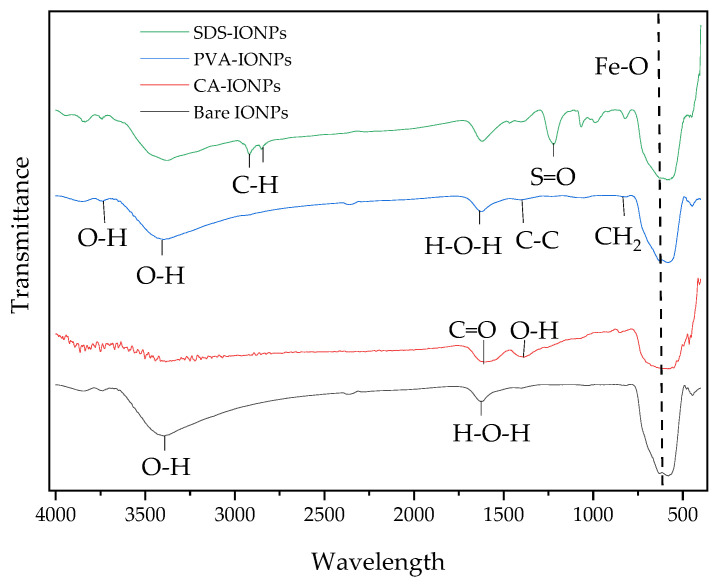
FTIR spectra of bare IONPs, CA-IONPs, PVA-IONPs, and SDS-IONPs.

FTIR spectroscopy was used to identify the type of functional groups present on the surface of the synthesized nanoparticles during the coating process. Figure 6 shows the FTIR spectrum of CA-IONPs, SDS-IONPs and PVA-IONPs. According to Figure 6, the peak between 500–800 cm^−1^ is ascribed to the stretching of Fe-O bonding in the bare IONPs and coated IONPs [3,5,22,26]. When the bare IONPs were coated with citric acid, two important features seemed to appear at 1389 cm^−1^ and 1610 cm^−1^. These were attributed to the OH group of the citric acid and asymmetric stretching of C=O vibration from the COOH group of the citric acid [14,15]. There was a small shift from ~1655 cm^−1^ to ~1630 cm^−1^ between the bare IONPs and CA-IONPs spectra due to the chemisorption of citrate ions onto the surface of IONPs, as reported in the literature [14,26]. The carboxylate species from the citric acid adsorbed onto the Fe atoms of the iron oxide surface portrayed a partial single bond character to the C=O bond [19]. Another narrow band was observed at ~3398 cm^−1^ for bare IONPs, but after the coating process, this band became broader. This could be due to the excellent bonding between the carboxylate from the citric acid with the IONPs and converting the IONPs into a hydrophilic nature [27].

FTIR was further extended to study the conjugation of PVA with IONPs. The FTIR spectrum shows the alcoholic O-H stretching band in the polymers matrix chain at ~3406 cm^−1^ and ~3726 cm^−1^ [3,28]. The other bonds at ~1626 cm^−1^ and ~1390 cm^−1^ were attributed to the H-O-H and C-C stretching bands, respectively. The CH_2_ low band appeared at ~819 cm^−1^; this could be ascribed to the weak PVA film [29]. All the important characteristic bands were identified, showing that PVA was successfully coated onto the surface of IONPs. The FTIR spectrum for IONPs coated with SDS showed a characteristic band at 2919 cm^−1^ and 2850 cm^−1^ due to the stretching vibrations of the C-H group of SDS. Furthermore, an absorption band was found at ~1215 cm^−1^, allocated to the stretching mode of S=O of the SDS. Based on the FTIR analysis, it was verified that IONPs were covered with the SDS [5].

### 3.3. Colloidal Stability Analysis of Synthesized Nanoparticles

**Scheme 1:**
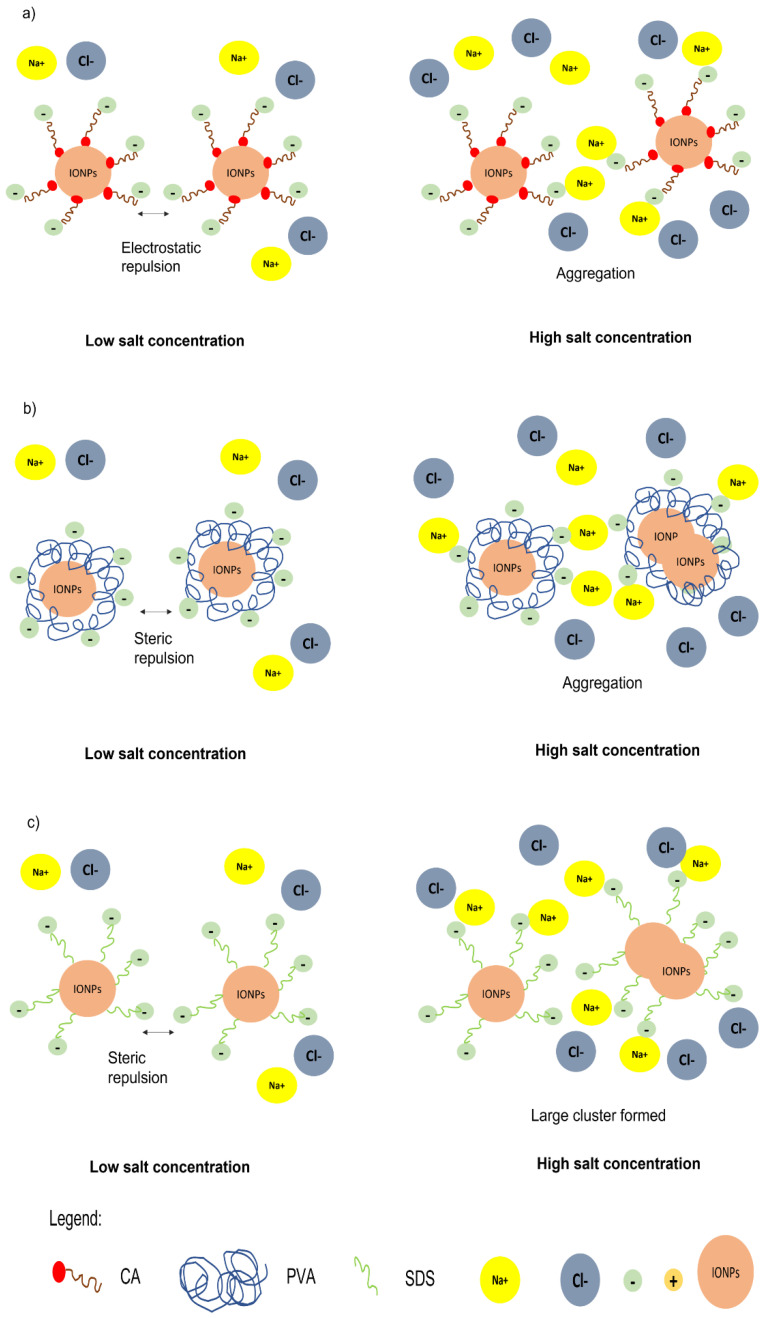
Interaction of (**a**) CA-IONPs, (**b**) PVA-IONPs, and (**c**) SDS-IONPs at low and high salt concentrations.

**Figure 7:**
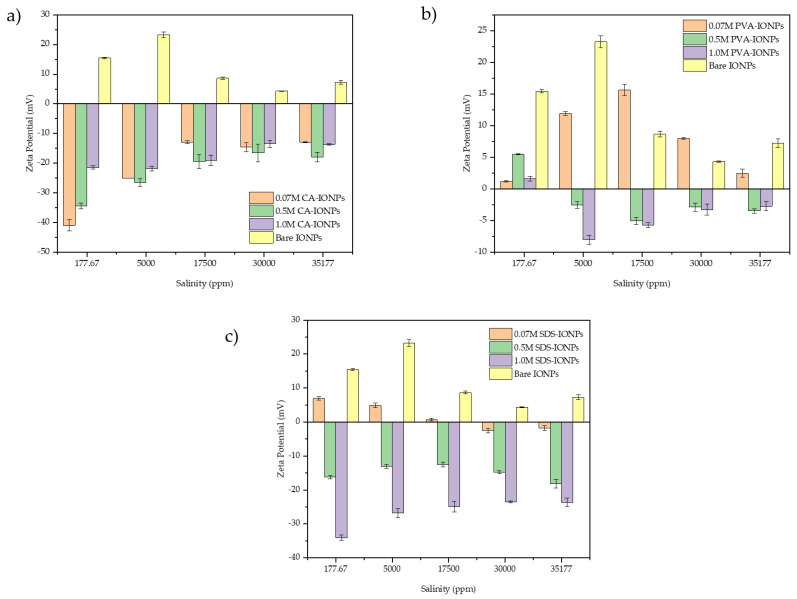
Zeta potential values of CA-IONPs, PVA-IONPs and SDS-IONPs under various molar ratios and salinities.

**Figure 8:**
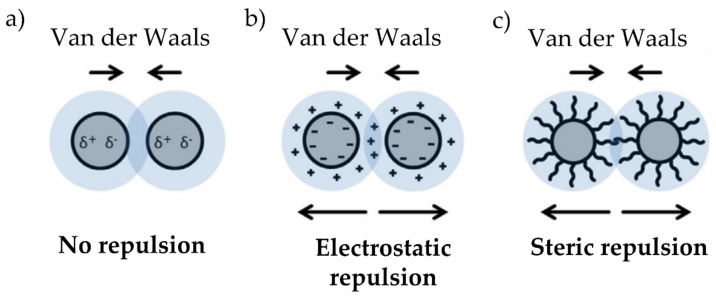
Schematic diagram of (**a**) No repulsion, (**b**) electrostatic repulsion and (**c**) steric repulsion. Published in 2020 by Elsevier under Creative Commons Attribution 4.0 (CC-BY-NC-ND 4.0) License.

**Figure 9:**
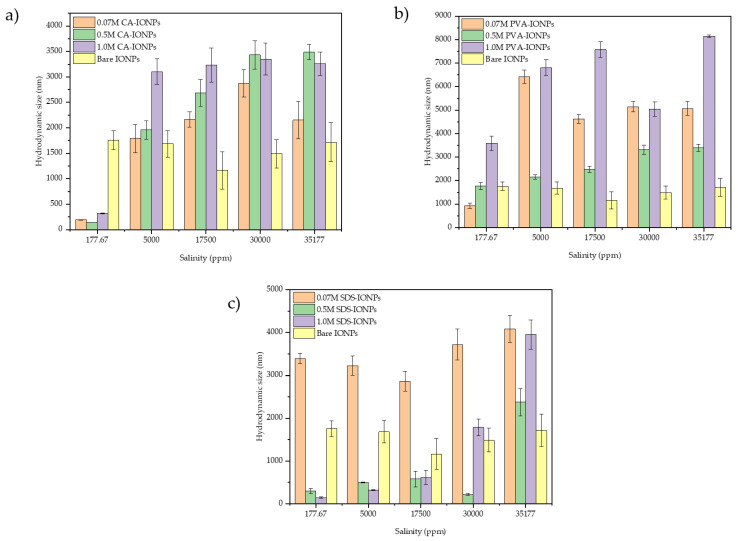
Hydrodynamic size of CA-IONPs, PVA-IONPs and SDS-IONPs under various molar ratios and salinities.

**Figure 10:**
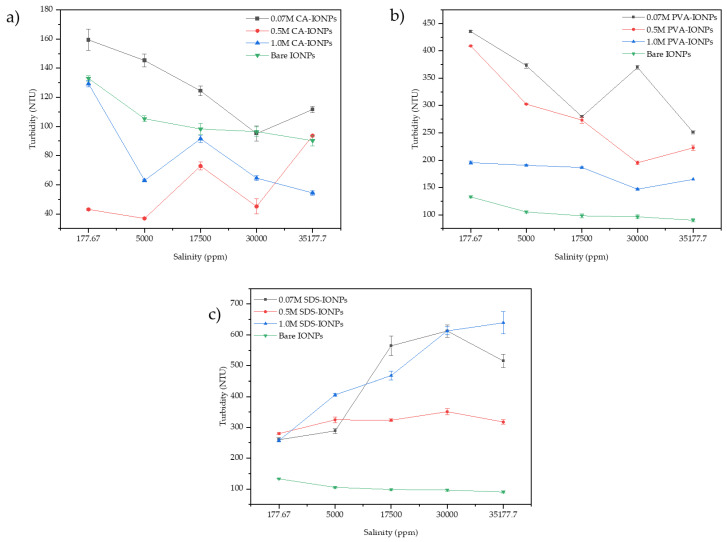
Turbidity values of CA-IONPs, PVA-IONPs and SDS-IONPs under various molar ratios and salinities.

The hydrodynamic size and zeta potential values of bare IONPs, CA-IONPs, PVA-IONPs and SDS-IONPs were measured using a Zetasizer and the turbidity was measured via a turbidity meter. Figure 7, Figure 8, Figure 9 and Figure 10 showed the results for the hydrodynamic size, zeta potential, and turbidity under various molar ratios (0.07 M, 0.5 M, 1.0 M) and different salinities (177.67 ppm, 5000 ppm, 17500 ppm, 30,000 ppm, and 35,177 ppm). In this study, the molar ratio of the coating agent and salinity were selected as one of the parameters, which was effective in controlling the zeta value and hydrodynamic size of the synthesized nanoparticles following the statements made in the related literature [14,19,24,25]. The interaction between coated IONPs at low and high salt concentration is illustrated in Scheme 1.

#### Zeta Potential Analysis

Figure 7a–c show the zeta potential values at various molar ratios and salinities for CA-IONPs, PVA-IONPs and SDS-IONPs, respectively. Based on Figure 7, the zeta values of bare IONPs increased from 15.43 mV to 23.23 mV when the salinity was increased to 5000 ppm. However, at above 5000 ppm, the zeta values started to reduce to 7.25 mV (around a 23% reduction). At the lower salinity, the pH of the salinity was nearly neutral. Therefore, the higher intensity of the surface charge may allow the bare IONPs to be more stable due to electrostatic repulsion forces [7]. The reduction in zeta value as the salinity was increased to 35177 ppm was due to the increase in monovalent ions (Na^+^) from the NaCl solution that makes the electrostatic interaction less favorable [7]. A higher salinity level indicates higher ionic strength in the medium [30].

After coating with CA onto the surface of IONPs, the colloidal stability was improved. This is based on the high zeta potential values recorded. The zeta potential values of 0.5 M CA-IONPs at 177.67 ppm, 5000 ppm, 17500 ppm, 30000 ppm and 35177 ppm were −34.4, −26.5, −19.4, −16.5 and −17.9 mV, respectively. The improved stability of the nanoparticles is due to the repelling forces among each other [16]. According to Figure 7a, at 177.67 ppm, 0.07 M of CA-IONPs have the highest zeta potential values of −40.9 mV, displaying a good colloidal stability and high electrostatic repulsive force. This clearly shows that 0.07 M CA-IONPs are adsorbed on the surface of the IONPs resulting in high negative surface charges, as reported in the previous literature [14,15]. In addition, the high negative zeta value further confirmed the presence of negatively charged carboxylate ions on the IONPs surface (Scheme 1). However, in the higher salinity region, the stability was compromised as zeta potential values reduced by 68% from −40.9 to −12.9 mV at 0.07 M CA-IONPs. This result suggests that the positively charged Na^+^ ions presence in the saline solution are bounded to the negatively charged CA-IONPs via electrostatic interactions [17] (Scheme 1).

The zeta potential for PVA-IONPs showed that under the higher molar ratio of PVA, the zeta value shifted to a more negative value (Figure 7b). The increase in the negative surface potential at 1.0 M PVA-IONPs can be ascribed to the ionization of the hydroxyl functional groups in the PVA. The negatively ionized hydroxyl groups in the PVA increase the zeta potential of 1.0 M PVA-IONPs and improve the electrostatic repulsion between the particles, providing more stability to the dispersion [7,17]. This result agrees with Tang et al. (2006) stating that the protective role of the PVA in preventing an agglomeration of IONPs is evident as the electrostatic repulsion between the polymer chains can hinder the agglomeration through steric hindrance [31] (Scheme 1). However, at a higher salinity, the negative zeta value at 1.0 M PVA-IONPs reduced from −8.0 mV to −2.7 mV. At the high salinity, the amount of positively charged ions also increased, dominating the negatively charged hydroxyl functional groups in the PVA. This therefore led to insufficient capping sites of PVA-IONPs resulting in the agglomeration [32]. 

Based on Figure 7c, 1.0 M SDS-IONPs have the highest zeta potential value across all salinity ranges. At a low salinity, the highest zeta value for 1.0 M SDS-IONPs was −34 mV, whereas in the high salinity region, the highest zeta potential value was 23.6 mV, which indicates a good stability. These results further proved the potential of SDS in improving thestability of IONPs in agreement with the Derjaguin, Verway, Landau, and Overbeek (DVLO) theory. This theory stated that the fundamental mechanism for dispersion stability comprises two types of dispersion forces: 1) electrostatic stabilization and 2) steric stabilization. Electrostatic stabilization can be based on the positively or negatively charged nanoparticles, formation of electrostatic double layer (EDL) by counter ions from the solution and electrostatic repulsion between the overlapping EDL (Figure 7) [33]. On the other hand, steric stabilization is based on the use of a bulky polymer chain or long-chained molecules on the particle’s surface, to form a steric protective layer that prevents direct particle contact and to create a steric repulsive force from the polymer chain, thereby increasing the surface charge (Figure 8) [5,16]. In this case, the SDS is an electrostatic stabilizer; therefore, the higher the amount of SDS coated on the IONPs’ surface, the more negatively charged the nanoparticles become. As the salinity started to increase, the electrolyte ions reduced the repulsion forces between the nanoparticles by neutralizing the surface charge of the particles [5].

### 3.4. Effect on the Hydrodynamic Size

Figure 9a–c present the hydrodynamic size for CA-IONPs, PVA-IONPs and SDS-IONPs at various molar ratios and salinities. The increase in the hydrodynamic size can be correlated with the zeta potential value. As the value of the zeta potential approaches zero under various salinities, the surface charge of the nanoparticles also decreases in intensity, which is related to the increase in the ionic strength. The results in Figure 7 show that some of the zeta potential values are near to zero. Therefore, neutralizing the nanoparticles’ surface charge may contribute to nanoparticle agglomeration, as indicated in Figure 9 as hydrodynamic size distribution. Compared to the size of the dried nanoparticles (Figure 3), the average diameter calculated in the solution was larger due to the Brownian motion in the solution and any slight fluctuation of light intensity during the measurement [16].

In Figure 9a, under salinities of 177.67 ppm and 35177 ppm, the average hydrodynamic size was 191.9 nm and 2152 nm of 0.07 M CA-IONPs, 138.2 nm and 3488.3 nm of 0.5 M CA-IONPs, 316 nm and 3259.3 nm of 1.0 M CA-IONPs, respectively. The huge difference in the hydrodynamic size indicated that salinity greatly induced the formation of larger agglomerates of nanoparticles attributed to the high ionic strength of the medium [30]. At 35177 ppm, when increasing the molar ratio from 0.07 M to 1.0 M the hydrodynamic size also increased from 2152 nm to 3259 nm. Although the size is considerable, the zeta potential values are also large at 35177 ppm of the NaCl solution. The smallest hydrodynamic size that could be observed in the lower salinity region was 138.2 nm for 0.5 M CA-IONPs. This condition corresponds to the high zeta potential value of −34.4 nm. The charge of citrate ions plays a vital role in the stability of the nanoparticles in water since CAs are soluble in water [34]. In a citrate, ion contains three carboxyl groups. The electric charge of these radical ions will create a repulsive force, hence making the nanoparticles more dispersed in water [34].

Compared with PVA-IONPs, the hydrodynamic size was larger and formed bigger agglomerates of up to 8300 nm (Figure 9b). The coating process using PVA produced larger agglomerates due to the interparticle crosslinking of the long polymer chains as the crosslinking agents [7]. The biggest hydrodynamic size is at 1.0 M PVA-IONPs, ranging from 930 nm to 8143 nm. These results are parallel with the low zeta potential value in Figure 7b, giving rise to van der Waal interparticle attraction and eventual coagulation and flocculation. The smallest hydrodynamic size was found at 0.07 M PVA-IONPs at 177.67 ppm (930 nm). However, this result does not indicate a high zeta potential value (1.2 mV). In this research, PVA was used to coat IONPs due to the creation of a steric barrier of the hydrophilic polymer. This is consistent with Di Marco et al. (2007), stating that if the polymer thickness of the coated IONPs is large enough, the van der Waals attraction between the particles is insignificant compared to the Brownian thermal energy. When the double layer is suppressed by the salt concentration, the access to each single chain of polymer is limited due to the overlap of the polymer layers. This will lead to a high total energy barrier and hence to a stronger repulsive force between the particles [30]. However, this study revealed a different result where, with increasing salinity, the negatively charged from the hydroxyl group tend to interact with the Na+ from the saline solution and be neutralized [3]. The high hydrodynamic size of PVA-IONPs and low zeta potential values under various molar ratios showed that the nanoparticles are prone to agglomeration, which indicates instability of the synthesized nanoparticles [7,35].

At 35177 ppm, 0.07 M SDS-IONPs has the highest hydrodynamic size of around 4087.7 nm. When there is an insufficient amount of surfactant to keep the particles separated, nanoparticle clusters could be formed [36]. Clustering of nanoparticles into large clusters could increase the hydrodynamic size of the nanoparticles. However, at 1.0 M SDS-IONP, the hydrodynamic size was smallest in most of the low salinity region (146 nm at 177.67 ppm, 319 nm at 5000 ppm, 615 nm at 17500 ppm), corresponding to the high zeta potential value at −34.3 mV, −26.8 mV, −24.9 mV, respectively. The hydrodynamic size started to increase as the salinity increased. SDS is an anionic surfactant (negatively charged); therefore, it has a strong affinity to positively charged ions such as Na^+^. As the salinity increased, the electrostatic interaction became stronger. At very high salinity values, the impact of extra electrolytes compressed the EDL around the nanoparticles, hence causing the hydrodynamic size to be bigger, which justifies the results found in Figure 9c and in the existing literature [37].

### 3.5. Effect on the Turbidity

Turbidity was used to measure the cloudiness of the solution. The value of the turbidity measured in a nephelometric turbidity unit (NTU) corresponds to the concentration of nanoparticles in the saline solution. The high value of turbidity indicates a good stability of the dispersion. Figure 10a–c show the turbidity for CA-IONPs, PVA-IONPs and SDS-IONPs at various molar ratios and salinities. Figure 10a,b showed that at 0.07 M has the highest turbidity value for the CA-IONP and PVA-IONPs compared to bare IONPs and SDS-IONPs. The high turbidity value for 0.07 M CA-IONPs (around 160 NTU) corresponds to the previous results that have a high zeta potential value and low hydrodynamic size; −40.9 mV and 191 nm, respectively. The high electrostatic repulsion force between the nanoparticles and the saline solution rendered more stability to the dispersion as the nanoparticles were less attracted to one another [38,39]. Therefore, less agglomeration occurs due to a low hydrodynamic size. Although 0.07 M CA-IONPs maintained a high turbidity value compared to other nanoparticles, they still experienced a reduction in turbidity value throughout the salinity range. This may be because the salt ions may have interacted with the synthesized nanoparticles.

On the other hand, PVA-IONPs showed a decreasing trend in terms of their turbidity results across the salinity. Although the range of turbidity values (165–435 NTU) was greater compared to CA-IONPs (54–160 NTU), the zeta potential value and hydrodynamic size do not support the turbidity findings. This could be due to PVA being water- soluble and comprising large amounts of hydroxyl groups that will form strong hydrogen bonds with the water molecules [40]. Therefore, the PVA-IONPs will remain soluble in the presence of low-salinity water.

Interestingly, for SDS-IONPs, 1.0 M has a higher turbidity value at a salinity of 177.67 ppm and 5000 ppm. Above 5000 ppm, 0.07 M of SDS-IONPs started to have a better turbidity value. The higher turbidity values at 1.0 M SDS-IONPs were 139 NTU and 63 NTU at 177.67 ppm and 5000 ppm, respectively. High salt concentration may cause instability in the dispersion as it can decrease the solubility of the surfactant in the aqueous phase. In general, 1.0 M of SDS-IONP showed a better consistency in terms of maintaining the high turbidity value across all salinity regions.

## 4. Conclusions

This paper demonstrates the effect of the molar ratio and salinity of PVA, SDS and CA on the zeta potential value, hydrodynamic size, and turbidity. Morphological and crystallographic further confirmed that coating processes were successfully attached to the surface of the IONPs. It was found that increasing the salt concentration caused instability to the synthesized PVA-IONPs, SDS-IONPs and CA-IONPs. It was reported that 0.07 M of CA-IONPs is the most stable at low salinities, resulting in the highest zeta potential value (−40.9 mV), smallest hydrodynamic size (192 nm) and best turbidity value (160 NTU). However, at high salt concentrations 1.0 M of SDS-IONPs have better stability as they are able to maintain a high zeta potential value (−23.63 mV), hydrodynamic radius (3955 nm) as well as the highest turbidity value (639.33 NTU). This work conclusively shows that CA and SDS are better candidates for the surface coating of IONPS as they can maintain a good stability in various salinity environments. In general, the presence of nanoparticles with excellent colloidal stability is not only able to disperse well in a suspension, but also has great potential in enhanced oil recovery application.

## Figures and Tables

**Table 1:** List of surface-coated iron oxide nanoparticles with their respective crystallite sizes.

Type of Surface Coating	Molar Ratio (M)	Crystallite Size from XRD (nm)	Size Measured from FE-SEM (nm)
CA-IONPs	0.07	9.726	34.9
CA-IONPs	0.5	12.399	33.9
CA-IONPs	1.0	10.643	42.7
SDS-IONPs	0.07	9.865	38.5
SDS-IONPs	0.5	8.595	33.9
SDS-IONPs	1.0	13.257	54
PVA -IONPs	0.07	9.635	34.3
PVA -IONPs	0.5	9.603	47.9
PVA -IONPs	1.0	9.969	52.2

## Data Availability

The raw/processed data required to reproduce these findings cannot be shared at this time as the data also form part of an ongoing Ph.D. study.
